# Therapeutic Cancer Vaccines in B-Cell Malignancies and Multiple Myeloma

**DOI:** 10.3390/vaccines14060473

**Published:** 2026-05-26

**Authors:** Vishrut Shah, Joseph Todd Martins

**Affiliations:** Hematology and Medical Oncology, The University of Texas Health Science Center at Tyler, Tyler, TX 75708, USA; joseph.martins@uttyler.edu

**Keywords:** cancer vaccines, multiple myeloma, lymphoma, B-cell, idiotype vaccines, neoantigen vaccines, dendritic cells, mRNA vaccines, hematologic neoplasms, immunotherapy

## Abstract

Therapeutic cancer vaccines represent a rational immunotherapeutic strategy aimed at inducing tumor-specific adaptive immune responses in patients with established malignancies. In contrast to prophylactic vaccines, these approaches must function within immunosuppressive tumor microenvironments characterized by antigenic heterogeneity, immune dysfunction, and dynamic tumor evolution. Effective vaccine design requires the integration of three essential components: the selection of appropriate tumor-associated or tumor-specific antigens, efficient delivery platforms that enable antigen presentation, and adjuvant systems that promote robust T-cell priming and expansion. Initial clinical investigations in B-cell malignancies and multiple myeloma demonstrated that idiotype-based vaccines can elicit tumor-specific immune responses. However, durable clinical benefit has been inconsistent, reflecting limitations in antigen selection, suboptimal immunogenicity, and tumor-mediated immune evasion. Over the past decade, advances in tumor genomics, next-generation sequencing, and immune monitoring have enabled the development of next-generation vaccine platforms, including dendritic cell-based approaches, personalized neoantigen vaccines, and mRNA-based technologies. Emerging evidence suggests that vaccine efficacy is highly dependent on disease context. Biologically favorable settings such as minimal residual disease (MRD) and post-transplant immune reconstitution provide reduced tumor burden and improved immune competence, thereby enhancing the likelihood of effective immune priming. In parallel, combination strategies incorporating immune checkpoint inhibitors, immunomodulatory agents, and cellular therapies are increasingly being explored to overcome tumor-induced immunosuppression. This review synthesizes current knowledge of therapeutic cancer vaccines in B-cell malignancies and multiple myeloma, with emphasis on immunologic mechanisms, antigen selection, vaccine platforms, and clinical evidence. We further propose a conceptual framework integrating tumor biology, immune context, and combination strategies to guide the rational development of next-generation vaccine therapies.

## 1. Introduction

The concept of immune-mediated tumor surveillance has evolved into a central principle of modern cancer biology and immunotherapy. Early experimental studies proposed that the immune system possesses the ability to recognize and eliminate malignant cells before clinically detectable disease develops [1]. Subsequent investigations demonstrated that defects in T-cell-mediated immunity and interferon signaling are associated with increased tumor incidence and accelerated disease progression, thereby supporting the importance of immune regulation in tumor control [2,3].

The relationship between tumor cells and host immunity is now best explained by the concept of cancer immunoediting, which consists of three interconnected phases: elimination, equilibrium, and escape [4,5,6]. During the elimination phase, innate and adaptive immune responses cooperate to recognize and eradicate transformed cells. In the equilibrium phase, immune pressure constrains tumor growth without complete eradication, resulting in the selection of increasingly resistant tumor clones. Ultimately, tumors may progress to the escape phase, characterized by antigen loss, impaired antigen presentation, induction of inhibitory immune checkpoint pathways, recruitment of suppressive immune cell populations, and establishment of highly immunosuppressive tumor microenvironments. These mechanisms collectively permit malignant cells to evade immune-mediated destruction and promote progressive disease development.

These observations established immunotherapy as a major therapeutic pillar in oncology [7,8,9]. Immunotherapeutic strategies can broadly be categorized into approaches that enhance pre-existing immune responses, such as immune checkpoint inhibition, and strategies that actively induce tumor-specific immunity, including therapeutic cancer vaccines [7,8,9,10]. Therapeutic vaccines aim to generate coordinated antigen-specific immune responses through the activation of dendritic cells, priming of CD4^+^ helper and CD8^+^ cytotoxic T lymphocytes, and establishment of durable immunologic memory. However, unlike prophylactic vaccines against infectious diseases, therapeutic cancer vaccines must function within environments characterized by immune dysfunction, chronic antigen exposure, and tumor-induced immune suppression.

B-cell malignancies and multiple myeloma represent particularly attractive settings for therapeutic vaccination because these diseases frequently express identifiable tumor-associated antigens and often achieve states of minimal residual disease following cytoreductive therapy. Early vaccine development focused predominantly on clonotypic immunoglobulin variable regions, known as idiotypes, which represent highly tumor-specific antigens unique to each malignant B-cell clone [10]. Additional target antigens subsequently investigated included cancer-testis antigens such as NY-ESO-1, lineage-associated antigens including CD19, CD20, and BCMA, and increasingly, tumor-specific neoantigens identified through genomic sequencing technologies [11,12,13,14,15].

Despite the strong biologic rationale and encouraging immunogenicity observed in early studies, therapeutic cancer vaccines historically produced inconsistent clinical benefit. Multiple factors contributed to these limitations, including tumor heterogeneity, antigenic drift, weak intrinsic antigen immunogenicity, impaired dendritic cell function, T-cell exhaustion, and highly suppressive tumor microenvironments [16,17,18,19]. Furthermore, individualized vaccine manufacturing approaches, particularly idiotype-based vaccines, introduced substantial logistical complexity and limited broader clinical implementation.

Over the past decade, major advances in next-generation sequencing, computational neoantigen prediction, immune profiling technologies, and vaccine platform development have substantially renewed translational interest in therapeutic cancer vaccination despite comparatively limited contemporary clinical datasets in B-cell malignancies. Personalized neoantigen vaccines, adaptive mRNA-based technologies, advanced immune monitoring, and increasingly sophisticated dendritic cell platforms now permit more individualized, scalable, and precision-based immunotherapeutic strategies capable of targeting dynamic tumor evolution and immune escape mechanisms [20,21,22,23,24,25,26].

Importantly, therapeutic vaccines are increasingly being integrated within broader precision immuno-oncology frameworks rather than functioning as isolated interventions. Rational combination approaches involving checkpoint inhibitors, immunomodulatory agents, epigenetic therapies, radiation therapy, and cellular therapies aim to simultaneously enhance antigen-specific immunity while reversing tumor-mediated immune suppression. Emerging evidence additionally suggests that treatment timing critically influences vaccine efficacy, with minimal residual disease states and post-transplant immune reconstitution representing particularly favorable biologic environments for vaccine-induced immune activation.

In this review, we summarize the immunologic principles underlying therapeutic cancer vaccination, critically evaluate major antigen-selection and vaccine-platform strategies, and review current clinical evidence in B-cell malignancies and multiple myeloma. Particular emphasis is placed on understanding the successes and limitations of earlier vaccine approaches, recent translational advances, and emerging precision immuno-oncology strategies likely to shape the future development of therapeutic cancer vaccines.

## 2. Immunologic Basis of Therapeutic Cancer Vaccines

The efficacy of therapeutic cancer vaccines depends upon coordinated interactions among antigen-presenting cells, CD4/CD8 T lymphocytes, cytokine signaling pathways, and the tumor microenvironment [20]. Successful vaccination requires efficient antigen processing and the presentation, activation and expansion of tumor-specific T cells, the trafficking of effector immune cells into tumor sites, and sustained immune function despite tumor-mediated suppression. Disruption at any stage of this process may significantly impair clinical efficacy and contribute to therapeutic resistance.

### 2.1. Antigen Processing and Presentation

Dendritic cells (DCs) are central mediators of antitumor immunity and represent the primary professional antigen-presenting cells responsible for the initiation of adaptive immune responses [21]. These cells capture tumor-derived antigens through phagocytosis, receptor-mediated endocytosis, and macropinocytosis before processing them into peptide fragments for presentation on major histocompatibility complex (MHC) molecules.

Antigen presentation on MHC class I molecules activates CD8^+^ cytotoxic T lymphocytes, whereas presentation on MHC class II molecules stimulates CD4^+^ helper T cells, which provide the cytokine support necessary for cytotoxic T-cell expansion, differentiation, persistence, and memory formation [22]. A particularly important mechanism in tumor immunology is cross-presentation, whereby exogenous tumor antigens are presented on MHC class I molecules, enabling the activation of cytotoxic T-cell responses against malignant cells. Because many tumor antigens originate extracellularly rather than through intracellular infection, cross-presentation is essential for effective vaccine-induced antitumor immunity.

The efficiency of antigen presentation depends upon dendritic cell maturation status, antigen load, co-stimulatory signaling, and the surrounding cytokine milieu. Importantly, immature dendritic cells may induce immune tolerance rather than immune activation, emphasizing the importance of adjuvant systems and innate immune stimulation in therapeutic vaccine design.

### 2.2. Co-Stimulation and T-Cell Activation

Effective T-cell activation requires both antigen recognition and co-stimulatory signaling. Interaction between the T-cell receptor and peptide-MHC provides antigen specificity, while secondary co-stimulatory pathways such as CD28-CD80/CD86 interactions are necessary for full T-cell activation and the prevention of anergy [23].

In the absence of adequate co-stimulation, T cells may become functionally inactive or undergo apoptosis, resulting in ineffective immune responses despite antigen exposure. Conversely, excessive or persistent antigen stimulation may promote T-cell exhaustion characterized by impaired proliferation, reduced cytokine production, and diminished cytotoxic function.

Activated CD8^+^ cytotoxic T lymphocytes mediate tumor-cell killing through several complementary mechanisms, including the release of perforin and granzymes, engagement of death receptors such as Fas (CD95), and secretion of cytokines including interferon-γ. These pathways induce apoptosis in malignant cells while simultaneously enhancing antigen presentation and amplifying local immune activation. CD4^+^ helper T cells additionally support the development of durable immune memory, tissue invasiveness and maintenance of long-term immune surveillance [20].

### 2.3. Immune Checkpoint Pathways

Immune checkpoint pathways play essential roles in regulating immune homeostasis and preventing excessive immune activation. Tumors frequently exploit these pathways to suppress antitumor immune responses and induce T-cell exhaustion [16,17,18,19].

Programmed death-1 (PD-1) is expressed on activated T cells and interacts with programmed death ligand-1 (PD-L1), expressed on tumor cells and antigen-presenting cells. This interaction suppresses T-cell proliferation, cytokine production, and cytotoxic activity. Similarly, cytotoxic T-lymphocyte-associated antigen-4 (CTLA-4) negatively regulates T-cell activation through competition with CD28 for binding to CD80/CD86 on antigen-presenting cells.

These inhibitory pathways substantially limit vaccine efficacy because vaccine-induced T cells may become rapidly suppressed within the tumor microenvironment. Consequently, therapeutic vaccines are increasingly being combined with immune checkpoint inhibitors to enhance the persistence and functionality of vaccine-induced immune responses.

### 2.4. Tumor Microenvironment and Immune Evasion

Despite effective antigen presentation and T-cell activation, tumors employ numerous strategies to evade immune-mediated destruction [16,17,18,19]. Malignant cells frequently downregulate antigen presentation machinery, reduce MHC expression, secrete immunosuppressive cytokines such as transforming growth factor-β and interleukin-10, and recruit regulatory T cells and myeloid-derived suppressor cells.

These mechanisms collectively create highly immunosuppressive tumor microenvironments characterized by impaired dendritic cell maturation, reduced T-cell activation, and progressive T-cell dysfunction. Chronic antigen exposure further promotes immune exhaustion and limits the durability of vaccine-induced immune responses.

Tumor heterogeneity and clonal evolution additionally contribute to immune escape through emergence of antigen-negative tumor subclones. These observations highlight why single-antigen vaccine approaches frequently produced inconsistent clinical benefit despite measurable immunogenicity.

### 2.5. The Cancer–Immunity Cycle

The cancer–immunity cycle provides a conceptual framework for understanding how therapeutic vaccines augment antitumor immunity [7]. This process begins with the release of tumor antigens following tumor-cell death, followed by antigen uptake and presentation by dendritic cells. Subsequent activation and expansion of tumor-specific T cells permits the trafficking of effector lymphocytes into tumor sites, where immune-mediated tumor-cell killing results in further antigen release and perpetuation of the cycle.

Therapeutic vaccines primarily aim to enhance antigen presentation and T-cell priming; however, durable clinical efficacy depends on the successful coordination of multiple steps within this broader immunologic process. Failure at any stage—including impaired antigen presentation, inadequate T-cell trafficking, immune checkpoint activation, or suppressive tumor microenvironments—may substantially limit vaccine efficacy.

### 2.6. Implications for Modern Vaccine Development

Advances in genomics, immune profiling, and computational neoantigen prediction have substantially improved understanding of tumor–immune interactions and enabled the development of increasingly individualized vaccine approaches. Modern therapeutic cancer vaccines are therefore increasingly being designed not as isolated interventions but rather as components of integrated immunotherapeutic strategies aimed at establishing durable immune surveillance and long-term disease control.

These insights have driven the evolution of therapeutic vaccine development from earlier static single-antigen approaches toward increasingly sophisticated strategies incorporating neoantigen targeting, adaptive mRNA-based platforms, immune checkpoint blockade, and rational combination immunotherapy.

## 3. Tumor Antigens in B-Cell Malignancies and Multiple Myeloma

The selection of appropriate tumor antigens is a critical determinant of vaccine efficacy because it directly influences the specificity, magnitude, and durability of antitumor immune responses. An ideal tumor antigen should demonstrate high tumor specificity, stable expression across malignant cell populations, strong immunogenic potential, and minimal expression on normal tissues. In B-cell malignancies and multiple myeloma, several antigen classes have been explored, each possessing distinct biologic advantages and translational limitations.

**Figure 1:**
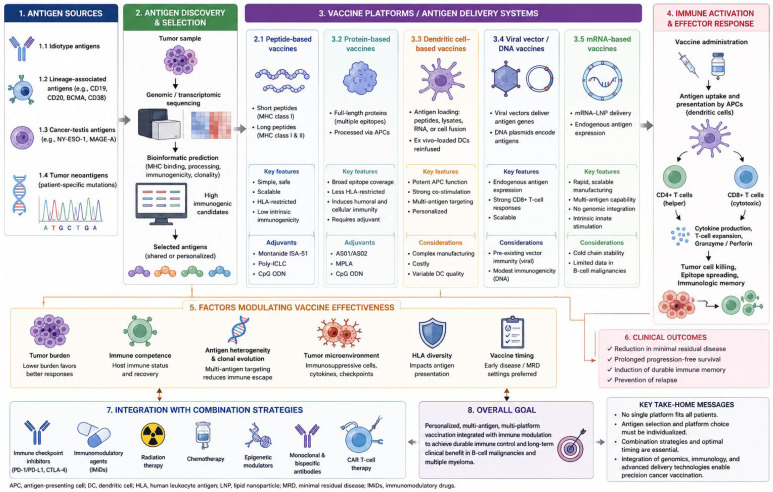
Conceptual framework of therapeutic cancer vaccine development and immune activation. The figure illustrates the conceptual framework of therapeutic cancer vaccine development in B-cell malignancies and multiple myeloma. Tumor-associated antigens and neoantigens identified through genomic and transcriptomic profiling are incorporated into multiple vaccine platforms including peptide-based, protein-based, dendritic cell-based, viral vector, DNA, and mRNA-based vaccines. Following antigen presentation by dendritic cells, activation of CD4^+^ helper and CD8^+^ cytotoxic T-cell responses promotes tumor-cell killing, epitope spreading, and development of immunologic memory. The figure additionally highlights major factors influencing vaccine efficacy, including tumor burden, immune competence, antigen heterogeneity, and tumor microenvironment–mediated immunosuppression, together with integration of combination immunotherapy strategies such as checkpoint inhibitors, immunomodulatory agents, radiation therapy, and cellular therapies.

To better contextualize therapeutic vaccine development within the broader cancer–immunity cycle, Figure 1 illustrates a generalized framework integrating tumor antigen identification, antigen presentation, T-cell priming, tumor-cell killing, and the development of immunologic memory. Although neoantigen-based strategies are emphasized, the conceptual principles represented in the figure are broadly applicable to multiple vaccine platforms including idiotype-based and shared tumor-associated antigen approaches.

### 3.1. Idiotype Antigens

Idiotype antigens were among the earliest and most extensively investigated vaccine targets in B-cell malignancies [10,24,25]. These antigens arise from the unique variable regions of immunoglobulins expressed by malignant B-cell clones and therefore represent highly tumor-specific targets unique to each patient’s disease. Because idiotype proteins are largely absent from normal tissues outside the malignant clone, they initially appeared to represent ideal vaccine antigens with minimal risk of off-target toxicity.

Early studies in follicular lymphoma and multiple myeloma demonstrated that idiotype vaccines could induce measurable humoral and cellular immune responses, thereby providing proof-of-principle that personalized tumor-specific vaccination could generate antitumor immunity in hematologic malignancies. Idiotype proteins can be processed and presented on both MHC class I and class II molecules, permitting coordinated activation of CD8^+^ cytotoxic T cells and CD4^+^ helper T cells.

Despite strong biologic rationale, several important limitations hindered broader clinical implementation of idiotype vaccines. Individualized vaccine production required patient-specific manufacturing processes that were labor-intensive, costly, and associated with substantial delays. In addition, idiotype antigens frequently demonstrated relatively weak intrinsic immunogenicity and therefore required conjugation to carrier proteins, adjuvant systems, or dendritic cell platforms to generate clinically meaningful immune responses. Tumor evolution and antigenic drift further limited the effectiveness of isolated single-antigen targeting approaches.

An important observation emerging from idiotype vaccine studies was the phenomenon of epitope spreading, in which an initial immune response directed against a specific idiotype subsequently expanded to include additional tumor-associated antigens [25]. This concept provided early evidence that therapeutic vaccination may induce broader immune remodeling beyond isolated antigen targeting and contributed to the development of modern multi-antigen and neoantigen-based strategies.

### 3.2. Lineage-Associated Antigens

Lineage-associated antigens including CD19, CD20, CD22, CD38, and B-cell maturation antigen (BCMA) are broadly expressed across B-cell malignancies and multiple myeloma and have demonstrated substantial therapeutic utility in monoclonal antibody therapies and CAR T-cell approaches [11,12,13,14,26]. Their relatively stable expression across malignant cell populations makes them attractive targets from the perspective of tumor coverage and clinical applicability.

However, unlike idiotype antigens, lineage-associated antigens are also expressed on normal B cells and plasma cells, thereby limiting tumor specificity and increasing the risk of off-target immune toxicity. Vaccine-induced immune responses targeting these antigens may therefore result in the depletion of normal immune cell populations and contribute to immunodeficiency and infectious complications.

In addition, many lineage-associated antigens represent self-antigens and are therefore subject to central immune tolerance mechanisms that reduce their intrinsic immunogenicity and limit the magnitude of vaccine-induced T-cell responses. Consequently, although lineage-associated antigens have achieved substantial success in antibody-based therapies and cellular immunotherapy, they have been less effective as isolated vaccine targets. Current strategies increasingly emphasize incorporating these antigens within broader multi-antigen or combination immunotherapeutic approaches.

### 3.3. Cancer-Testis Antigens

Cancer-testis antigens (CTAs), including NY-ESO-1, MAGE family proteins, and PRAME, represent a distinct class of tumor-associated antigens characterized by restricted expression in immune-privileged tissues such as the testis together with aberrant expression in malignant cells [27]. Because these antigens are largely absent from normal somatic tissues, they may evade central immune tolerance and generate potent humoral and cellular immune responses.

Clinical studies involving NY-ESO-1 demonstrated coordinated activation of CD4^+^ helper and CD8^+^ cytotoxic T-cell responses together with the induction of antigen-specific antibodies, thereby validating the immunogenic potential of cancer-testis antigens [28,29]. These findings generated substantial interest in developing CTA-targeted vaccines for hematologic malignancies and solid tumors.

However, the expression of cancer-testis antigens is often heterogeneous both between patients and within individual tumors. This variability limits the effectiveness of isolated single-antigen targeting and may facilitate immune escape through the emergence of antigen-negative tumor subclones. Consequently, modern therapeutic strategies increasingly incorporate cancer-testis antigens within broader multi-antigen vaccine constructs designed to improve immune coverage and reduce the likelihood of immune evasion.

### 3.4. Tumor Neoantigens and Evolving Antigen Targets

The limitations associated with earlier single-antigen vaccine strategies have stimulated increasing interest in broader and more individualized antigen-targeting approaches. Tumor heterogeneity, antigenic drift, and immune escape frequently limit the durability of immune responses directed against shared tumor-associated antigens. Consequently, therapeutic cancer vaccine development has progressively shifted toward the identification of highly immunogenic and tumor-specific neoantigens generated through somatic mutations [20,21,22].

Neoantigens are mutation-derived peptides uniquely expressed by malignant cells and absent from normal tissues. Because these antigens are not subject to central immune tolerance, they may induce stronger and more selective T-cell responses compared with conventional tumor-associated antigens. Advances in next-generation sequencing and computational epitope prediction have enabled the increasingly precise identification of patient-specific neoantigen targets suitable for individualized vaccine development [20,21,22].

In addition to neoantigens, several other antigen categories continue to play important roles in therapeutic vaccine research. Idiotype antigens provided the earliest proof-of-principle for personalized vaccination strategies in B-cell malignancies and multiple myeloma, while shared tumor-associated antigens such as NY-ESO-1, MAGE family proteins, survivin, WT1, BCMA, and CD38 remain attractive because of their broader applicability across patient populations [13,14,15,26,27,28,29,30,31,32,33].

Importantly, each antigen category possesses distinct advantages and translational limitations related to tumor specificity, immunogenicity, manufacturing complexity, and susceptibility to immune escape. These considerations increasingly influence antigen selection, vaccine platform design, and rational integration with complementary immunotherapeutic strategies.

The major antigen classes currently investigated in therapeutic cancer vaccination are summarized in Table 1.

### 3.5. Conceptual Integration: From Antigen Selection to Personalized Vaccination

The process of therapeutic cancer vaccine development is illustrated in Figure 1, which integrates tumor antigen identification, vaccine platform selection, immune activation, and factors influencing clinical efficacy within the cancer–immunity cycle. As shown in the diagram, tumor-associated antigens and neoantigens are identified through genomic and transcriptomic profiling and subsequently incorporated into vaccine platforms including peptide-based vaccines, protein vaccines, dendritic cell vaccines, viral vector or DNA-based systems, and mRNA constructs. Following antigen presentation by dendritic cells, activation of CD4^+^ helper and CD8^+^ cytotoxic T-cell responses promotes tumor-cell killing, epitope spreading, and development of durable immunologic memory. The figure additionally highlights the influence of tumor burden, immune competence, antigen heterogeneity, and tumor microenvironment-mediated immunosuppression, together with the role of combination immunotherapy strategies in enhancing vaccine efficacy.

Importantly, the effectiveness of this process is influenced by multiple factors, including tumor burden, immune competence, antigen heterogeneity, and the presence of immunosuppressive mechanisms within the tumor microenvironment. These observations reinforce the concept that antigen selection alone is insufficient for effective therapeutic vaccination. Rather, successful vaccine strategies require coordinated integration of appropriate antigen selection, scalable delivery platforms, immune modulation, and rational combination therapies.

Recent advances in genomics, computational neoantigen prediction, immune profiling, and mRNA vaccine technology have substantially accelerated the development of increasingly personalized immunotherapeutic strategies [34,35,36,37,38,39,40,41,42,43,44]. Consequently, therapeutic cancer vaccines are progressively evolving from relatively static single-antigen approaches toward adaptive precision immuno-oncology platforms capable of dynamically responding to tumor evolution and immune escape mechanisms.

## 4. Vaccine Platforms and Antigen Delivery Systems

While antigen selection determines the specificity of the immune response, the vaccine platform critically influences its magnitude, quality, durability, and translational feasibility. Effective therapeutic vaccination requires the coordinated integration of three major components: antigen selection, delivery platform, and immune-stimulatory adjuvant systems. The delivery platform governs antigen uptake, dendritic cell activation, intracellular antigen processing, and the balance between CD4^+^ helper and CD8^+^ cytotoxic T-cell responses.

A major challenge in therapeutic cancer vaccine development is that most tumor-associated antigens possess limited intrinsic immunogenicity. Consequently, successful vaccine platforms must not only deliver antigens efficiently but also provide sufficient immunostimulatory signaling capable of overcoming immune tolerance, T-cell exhaustion, and tumor-induced immunosuppression. Over the past decade, advances in genomics, molecular engineering, and immune profiling have substantially accelerated the development of increasingly sophisticated vaccine technologies. These approaches have evolved from relatively simple peptide-based platforms toward more adaptive and personalized systems including dendritic cell vaccines, viral vectors, and mRNA-based neoantigen vaccines.

### 4.1. Peptide-Based Vaccines

Peptide-based vaccines consist of short or long synthetic peptides corresponding to defined tumor-associated antigenic epitopes and are typically administered together with adjuvants to enhance immunogenicity [30,31]. Short peptides are presented directly on MHC class I molecules and preferentially activate CD8^+^ cytotoxic T lymphocytes. In contrast, long peptides require processing by professional antigen-presenting cells and may subsequently be presented on both MHC class I and class II molecules, thereby inducing broader immune responses involving both CD4^+^ helper and CD8^+^ cytotoxic T cells.

Several peptide vaccine studies targeting antigens such as WT1, survivin, RHAMM, and NY-ESO-1 demonstrated the induction of antigen-specific T-cell responses in hematologic malignancies [28]. However, durable clinical efficacy remained inconsistent. One major limitation involves human leukocyte antigen (HLA) restriction, which limits applicability to subsets of patients expressing compatible MHC alleles. In addition, many peptide vaccines demonstrate relatively weak intrinsic immunogenicity and therefore require potent adjuvant systems to generate clinically meaningful immune activation.

Despite these limitations, peptide vaccines remain attractive because of their relative simplicity, favorable safety profile, scalability, and compatibility with combination immunotherapy approaches. Modern peptide vaccine strategies increasingly incorporate multiple epitopes simultaneously to improve immune breadth and reduce the likelihood of immune escape through antigen loss.

### 4.2. Protein-Based Vaccines

Protein-based vaccines utilize full-length tumor antigens that are processed by antigen-presenting cells into multiple peptide epitopes for presentation on both MHC class I and class II molecules [33]. This broader epitope repertoire reduces HLA restriction and increases the likelihood of eliciting coordinated humoral and cellular immune responses across diverse patient populations.

An important advantage of protein-based vaccination is the ability to induce both antibody-mediated and T-cell-mediated immunity, as demonstrated in studies involving cancer-testis antigens such as NY-ESO-1 [29]. Because multiple epitopes are generated during antigen processing, protein vaccines may additionally facilitate epitope spreading and broader immune recognition.

However, similar to peptide-based vaccines, protein vaccines often lack sufficient intrinsic immunogenicity and therefore require potent adjuvant systems to activate dendritic cells effectively. In addition, protein processing efficiency and variability in antigen presentation may influence the magnitude and reproducibility of immune response.

### 4.3. Dendritic Cell-Based Vaccines

Dendritic cell (DC)-based vaccines directly exploit the biologic function of professional antigen-presenting cells to optimize immune activation. This approach involves the isolation of autologous dendritic cells, ex vivo loading with tumor antigens through peptides, tumor lysates, RNA, apoptotic cells, or cell-fusion techniques, followed by reinfusion into the patient [22,32].

The principal advantage of dendritic cell vaccination lies in its ability to provide both efficient antigen presentation and robust co-stimulatory signaling, thereby overcoming several limitations associated with passive in vivo antigen delivery. DC-based vaccines additionally permit patient-specific customization and multi-antigen targeting.

Clinical studies in follicular lymphoma and multiple myeloma demonstrated measurable tumor-specific T-cell activation and evidence of biologic activity using dendritic cell vaccine approaches [45,46]. In particular, dendritic cell/myeloma fusion vaccines generated expansion of tumor-reactive T-cell populations and showed encouraging activity in post-autologous stem cell transplant settings.

Despite strong immunologic rationale, broader clinical translation has remained limited by logistical complexity, high manufacturing costs, variability in dendritic cell quality, and lack of standardization in vaccine production. These challenges contributed to inconsistent clinical outcomes despite clear evidence of immunologic activity.

Importantly, emerging evidence suggests that dendritic cell vaccines may achieve the greatest efficacy in low-disease-burden settings or when combined with checkpoint inhibitors and immunomodulatory therapies capable of enhancing T-cell persistence and reversing tumor-mediated immune suppression.

### 4.4. Viral Vector and Dna-Based Vaccines

Viral vector vaccines utilize genetically engineered viral systems to deliver antigen-encoding sequences into host cells, thereby enabling endogenous antigen expression and presentation through MHC class I pathways. This mechanism effectively mimics intracellular infection and promotes strong CD8^+^ cytotoxic T-cell responses.

DNA vaccines employ plasmid DNA encoding tumor antigens, which is taken up by host cells and translated into protein. These platforms offer several theoretical advantages including stability, scalability, ease of production, and sustained antigen expression.

However, both approaches possess important limitations. Pre-existing immunity to viral vectors may reduce efficacy, particularly with repeated dosing, while DNA vaccines have historically demonstrated relatively weak immunogenicity in clinical settings. Consequently, their translational impact in hematologic malignancies has thus far remained limited.

Nevertheless, advances in vector engineering, electroporation techniques, and adjuvant development continue to improve the potential applicability of these platforms.

### 4.5. mRNA-Based Vaccines

mRNA -based vaccines represent one of the most important technological advances in modern cancer immunotherapy [38,39,40]. These platforms utilize synthetic messenger RNA-encoding tumor antigens, which are delivered into host cells and translated into protein, thereby enabling endogenous antigen presentation through both MHC class I and class II pathways.

Several important advantages distinguish mRNA vaccines from earlier platforms. These include rapid and scalable manufacturing, the absence of genomic integration risk, the simultaneous encoding of multiple antigens, and dynamic adaptability to evolving tumor genomics. In addition, mRNA molecules possess intrinsic immunostimulatory properties capable of enhancing dendritic cell activation and innate immune signaling.

Recent clinical studies involving personalized neoantigen mRNA vaccines demonstrated robust and durable T-cell responses, particularly when combined with immune checkpoint inhibition [36,41,44]. These findings substantially renewed interest in therapeutic cancer vaccination and established mRNA platforms as leading candidates for personalized immunotherapy.

Although clinical experience in B-cell malignancies and multiple myeloma remains relatively limited, ongoing studies continue to evaluate the efficacy of mRNA-based neoantigen vaccines within these disease settings. Given the ongoing somatic hypermutation and clonal evolution characteristic of many B-cell malignancies, mRNA platforms may prove particularly well-suited for adaptive personalized vaccine strategies.

### 4.6. Integration of Antigen and Platform Selection: A Translational Framework

A critical insight emerging from both preclinical and clinical studies is that antigen selection and vaccine platform design are fundamentally interdependent. The effectiveness of therapeutic cancer vaccination depends not solely on identification of target antigens but also on the ability of vaccine platforms to efficiently deliver these antigens, activate antigen-presenting cells, and generate durable cellular immune responses. Consequently, modern therapeutic vaccine development increasingly emphasizes the coordinated optimization of antigen selection, delivery technology, immune modulation, and treatment timing.

As illustrated conceptually in Figure 1, therapeutic cancer vaccination involves the coordinated integration of tumor-associated antigen identification, vaccine platform selection, dendritic cell activation, and downstream T-cell-mediated immune responses within the broader cancer–immunity cycle. Multiple vaccine platforms including peptide-based vaccines, dendritic cell vaccines, viral vectors, and mRNA-based delivery systems are currently being investigated to optimize antigen presentation and promote durable CD4^+^ helper and CD8^+^ cytotoxic T-cell activation.

Importantly, the effectiveness of these processes is influenced by multiple biologic variables including tumor burden, immune competence, antigen heterogeneity, and the presence of immunosuppressive mechanisms within the tumor microenvironment. High tumor burden and chronic antigen exposure may promote T-cell exhaustion and impair vaccine responsiveness, whereas minimal residual disease settings following cytoreductive therapy or autologous stem cell transplantation may provide more favorable immunologic conditions for vaccine-induced immune expansion and long-term immune surveillance.

These observations reinforce the concept that antigen selection alone is insufficient for effective therapeutic vaccination. Rather, successful immunotherapeutic strategies require the integration of highly immunogenic antigens with scalable and adaptive vaccine platforms, together with the rational incorporation of complementary immunomodulatory approaches. Increasingly, vaccine efficacy is being enhanced through combination with checkpoint inhibitors, immunomodulatory agents, epigenetic therapies, radiation therapy, and cellular immunotherapies.

Collectively, these developments support a major conceptual transition from isolated vaccine design toward integrated precision immuno-oncology strategies in which antigen selection, vaccine platform technology, immune modulation, and longitudinal immune monitoring are optimized in a coordinated and adaptive manner [34,35,36,37,38,39,40,41,42,43,44,47,48,49,50,51,52].

Collectively, these observations demonstrate that therapeutic vaccine efficacy depends not only on antigen selection but also on appropriate platform design, scalability, immune activation, and integration with complementary immunotherapeutic strategies. The principal vaccine platforms currently investigated in B-cell malignancies and multiple myeloma, together with their mechanisms, advantages, and translational limitations, are summarized in Table 2.

## 5. Therapeutic Vaccines in B-Cell Malignancies

B-cell malignancies represent one of the earliest and most extensively investigated settings for therapeutic cancer vaccination because these diseases frequently express identifiable tumor-associated antigens and often achieve states of minimal residual disease following therapy. Indolent lymphomas, particularly follicular lymphoma, provided the strongest early evidence supporting the biologic feasibility of vaccine-induced antitumor immunity. However, clinical outcomes have varied substantially across disease subtypes because of differences in tumor biology, immune dysfunction, disease kinetics, and treatment context. Key clinical trials evaluating therapeutic cancer vaccines in B-cell malignancies and multiple myeloma are summarized in Table 3.

### 5.1. Follicular Lymphoma

Follicular lymphoma is the most extensively studied model for therapeutic cancer vaccination and provides the strongest clinical evidence. Its indolent progression, consistent expression of clonotypic immunoglobulin idiotype antigens, and achievable MRD states make it ideal for vaccine investigation.

Kwak and associates (1992) provided initial proof-of-concept in nine patients with B-cell non-Hodgkin lymphoma in MRD: tumor-derived idiotype protein conjugated to keyhole limpet hemocyanin (KLH) with adjuvant induced sustained idiotype-specific immune responses in seven of nine patients [10]. Hsu’s group (1996) advanced the approach using autologous dendritic cells pulsed ex vivo with the idiotype antigen; all four treated patients showed immune responses, with three achieving clinical benefit [46].

A pivotal 1997 study by Hsu, Caspar, and Czerwinski in 41 patients with low-grade B-cell lymphoma demonstrated that anti-idiotype responders (approximately half) experienced significantly prolonged remissions and superior survival compared to non-responders, establishing immunologic response as predictive of clinical outcome [55]. Bendandi and colleagues (1999) demonstrated the vaccine’s ability to eradicate MRD: eight of eleven follicular lymphoma patients with detectable residual disease achieved molecular remission using idiotype vaccination with GM-CSF [25]. Timmerman’s group (2002) treated 35 patients with dendritic cell-based idiotype vaccination; 22 showed objective tumor regression, and notably, six initially non-responsive patients subsequently responded to booster vaccination, indicating that repeated antigen exposure can overcome initial immune tolerance [53].

Phase II studies confirmed the clinical significance of vaccine-induced immunity. Rodríguez-Calvillo and colleagues (2006) vaccinated 25 patients in second complete remission, with 20 showing strong immune responses; responders experienced second remissions of 20–51 months versus 8–13 months for non-responders [56]. Redfern and colleagues (2006) documented objective responses and prolonged disease stabilization with idiotype-KLH vaccination, maintaining an excellent safety profile [57].

Researchers subsequently explored multi-antigen approaches. Di Nicola and colleagues (2009) used dendritic cells loaded with whole tumor lysate presenting multiple antigens, yielding objective responses and disease stabilization in numerous patients [58]. A key Phase III trial by Schuster, Neelapu, and Gause (2011) [54] in 177 patients demonstrated that despite initial small survival benefit (23 vs. 20 months), manufacturing delays prevented many patients from receiving the vaccine. In vaccinated patients who received treatment, disease-free survival was substantially longer (44 vs. 30 months), highlighting both the potential and practical challenges of personalized vaccination [54]. Navarrete and colleagues (2011) explored pre-cytoreduction vaccination with autologous recombinant idiotype Fab fragment and MF59 adjuvant, achieving median progression-free survival of four years with cellular immune responses correlating more strongly with outcomes than humoral responses [59].

Collectively, these studies establish follicular lymphoma as the gold standard model for therapeutic cancer vaccination. The absence of regulatory approval reflects logistical and manufacturing challenges rather than lack of biologic activity, providing definitive proof-of-principle that personalized tumor-specific vaccination can induce clinically meaningful immune responses.

### 5.2. Diffuse Large B-Cell Lymphoma (DLBCL)

DLBCL presents a distinct landscape for vaccination: rapid growth, large tumor burden, and immediate treatment need limit the applicability of vaccine strategies requiring time for immune response development. Additionally, frontline rituximab-based chemoimmunotherapy yields high cure rates, and CAR T-cell therapy substantially improves relapsed outcomes, raising the therapeutic bar for vaccine approaches.

Initial investigations demonstrated the dendritic cell-based vaccine’s capacity to stimulate tumor-specific T-cell responses in aggressive lymphomas [58]. However, these studies lacked disease-specificity and failed to demonstrate consistent or lasting clinical benefit in DLBCL specifically. Compared with indolent lymphomas, DLBCL may possess greater neoantigen-generating potential because of its increased genomic instability and higher mutational burden in selected molecular subtypes. These features may improve immunogenicity and provide opportunities for personalized neoantigen-directed vaccine strategies. However, the rapid clinical kinetics of aggressive lymphoma frequently limit the time available for individualized vaccine manufacturing and immune-priming, thereby complicating therapeutic implementation. Consequently, future approaches will likely emphasize the integration of vaccination with checkpoint inhibition, cellular therapies, and post-cytoreductive MRD-directed treatment strategies to optimize vaccine responsiveness and the durability of immune control. Promising directions include leveraging DLBCL’s elevated mutational burden to generate immunogenic neoantigens and exploiting somatic hypermutation-driven antigenic diversity through personalized neoantigen vaccine strategies. Future approaches will likely integrate vaccination with established modalities in MRD and post-transplant settings, potentially combined with checkpoint inhibitors to mitigate T-cell exhaustion.

### 5.3. Chronic Lymphocytic Leukemia (CLL)

CLL presents a particularly challenging environment due to significant immune dysfunction: dendritic cell maturation deficiency impairs antigen presentation, T-cell exhaustion limits cytotoxic capacity, hypogammaglobulinemia and expanded regulatory T-cell populations create profound immunosuppression. These factors impede vaccine efficacy despite adequate antigen presentation.

Dendritic cell-based vaccines employing autologous leukemia cells have elicited antigen-specific immune responses, validating the feasibility of vaccination [60]. Peptide-based vaccines targeting WT1 and survivin similarly generate measurable T-cell responses [61], though magnitude and persistence often prove inadequate. This underscores a fundamental principle: effective vaccination requires both antigen presentation and functional host immunity.

Recent developments offer promise: bruton tyrosine kinase inhibitors partially restore immune function in CLL by enhancing T-cell activity and reducing immunosuppressive signaling. Future strategies will emphasize combination approaches integrating vaccination with immune-modulating therapies to overcome CLL’s immunosuppressive microenvironment.

### 5.4. Mantle Cell Lymphoma (MCL)

MCL vaccines have received limited investigation. The disease features cyclin D1 overexpression, a severe clinical course, and frequent relapses. Early studies examining idiotype-based and dendritic cell-based approaches demonstrated immune response induction [24], though consistent clinical benefit remains unestablished.

As with other B-cell malignancies, vaccination timing proves critical: MRD and post-transplant settings offer the most favorable contexts, with reduced tumor burden and immune reconstitution enhancing the therapy’s effectiveness. With the emergence of Bruton tyrosine kinase inhibitors and cellular therapies, focus has shifted toward combination strategies. Vaccination may consolidate responses from these modalities and maintain long-term disease control.

### 5.5. Translational Insights and Evolving Principles from B-Cell Malignancy Vaccine Studies

Studies across B-cell malignancies have revealed several important biologic and translational principles that continue to shape modern therapeutic cancer vaccine development [10,25,46,53,54,58]. First, disease biology strongly influences vaccine responsiveness. Indolent diseases such as follicular lymphoma provide more favorable immunologic environments than rapidly progressive malignancies because slower tumor kinetics permit sufficient time for immune priming and T-cell expansion [25,53,54].

Second, tumor burden consistently emerges as a major determinant of vaccine efficacy. Clinical studies repeatedly demonstrated improved outcomes when vaccines were administered in minimal residual disease settings characterized by lower tumor burden and reduced immunosuppressive signaling [10,25,47,51,54].

Third, adequate host immune competence is essential for successful vaccination. Diseases associated with profound immune dysfunction, particularly chronic lymphocytic leukemia, present substantial barriers to effective immune activation unless combined with therapies capable of restoring immune function [16,17,18,19,60,61].

Importantly, these studies also highlighted the limitations of earlier single-antigen vaccine approaches. Tumor heterogeneity, clonal evolution, and antigenic drift frequently permitted immune escape through the emergence of antigen-negative subclones [5,6,16,17,18,19]. These observations strongly support the development of multi-antigen and personalized neoantigen-based strategies [34,35,36,41,42,43,44].

Finally, clinical trials underscored the major translational challenges associated with individualized vaccine manufacturing. The logistical complexity, prolonged production timelines, and scalability limitations of idiotype-based vaccines significantly hindered broader clinical implementation despite encouraging biologic activity [24,25,54].

Collectively, these findings support the evolving view that therapeutic cancer vaccines are unlikely to achieve maximal efficacy as isolated interventions. Instead, their future role will likely involve integration within broader immunotherapeutic frameworks combining vaccination with checkpoint inhibition, immunomodulatory agents, cellular therapies, and precision immune monitoring [16,17,18,19,38,39,40,41,42,43,44,47,48].

## 6. Therapeutic Cancer Vaccines in Multiple Myeloma

Multiple myeloma (MM) represents an attractive but uniquely challenging target for therapeutic vaccination. Several biologic features support vaccine development, including the presence of identifiable tumor-associated antigens, prolonged disease course, and the ability to achieve minimal residual disease following induction therapy and autologous stem cell transplantation (ASCT). In addition, periods of post-transplant immune reconstitution may provide particularly favorable environments for vaccine-induced T-cell expansion and memory formation.

However, multiple myeloma is also associated with profound immune dysregulation involving impaired dendritic cell function, T-cell exhaustion, the expansion of regulatory T cells, suppressive cytokine networks, and a highly immunosuppressive bone marrow microenvironment [62]. These mechanisms substantially limit spontaneous antitumor immunity and contribute to the inconsistent clinical efficacy observed with earlier vaccine approaches.

### 6.1. Idiotype-Based Vaccines

Initial vaccine studies in multiple myeloma focused predominantly on idiotype antigens derived from patient-specific monoclonal immunoglobulins. Similar to follicular lymphoma, these clonotypic proteins represented highly tumor-specific targets with minimal risk of off-target toxicity.

Early clinical trials demonstrated that idiotype vaccination could induce measurable humoral and cellular immune responses in subsets of patients [24,55]. However, durable clinical responses remained limited, particularly in patients with active or advanced disease burden. One major challenge involved the relatively weak intrinsic immunogenicity of idiotype proteins, which frequently required conjugation to carrier proteins or incorporation into dendritic cell platforms to enhance antigen presentation and T-cell activation.

Subsequent studies increasingly demonstrated that treatment timing critically influenced outcomes. Vaccination following cytoreductive therapy or ASCT appeared more effective than administration during progressive disease because the lower tumor burden and partial immune recovery permitted improved T-cell priming and expansion [47,51].

Despite encouraging immunologic activity, idiotype vaccines did not achieve widespread clinical implementation because of individualized manufacturing requirements, production delays, and inconsistent clinical efficacy.

### 6.2. Dendritic Cell-Based Vaccine Strategies

Dendritic cell (DC)-based vaccines are among the most extensively investigated immunotherapeutic approaches in multiple myeloma. These strategies utilize autologous dendritic cells loaded ex vivo with tumor antigens, tumor lysates, RNA, apoptotic cells, or fusion products to optimize antigen presentation and co-stimulatory signaling [24,36].

One particularly promising strategy involved the fusion of autologous myeloma cells with dendritic cells, thereby enabling the simultaneous presentation of a broad repertoire of tumor antigens while preserving efficient antigen-presenting function. Rosenblatt and colleagues demonstrated that dendritic cell/myeloma fusion vaccines could induce expansion of tumor-reactive T-cell populations and generate measurable clinical responses, particularly in post-ASCT settings [45].

Post-transplant immune reconstitution appears especially favorable for dendritic cell vaccination because the reduced tumor burden and recovering immune populations may enhance vaccine responsiveness. Several studies demonstrated the expansion of myeloma-specific T-cell populations together with prolonged disease stabilization following vaccination after ASCT [47].

However, durable responses remained inconsistent because of persistent immune suppression within the bone marrow microenvironment, antigenic heterogeneity, and progressive T-cell exhaustion. Consequently, current strategies increasingly emphasize combining dendritic cell vaccines with checkpoint inhibitors, immunomodulatory drugs, or other immune-enhancing therapies.

### 6.3. Peptide and Shared Antigen Vaccines

Several peptide-based vaccines targeting shared tumor-associated antigens including WT1, survivin, RHAMM, MUC1, and NY-ESO-1 have been investigated in multiple myeloma [39,40,41]. These antigens are variably expressed on malignant plasma cells and are capable of inducing antigen-specific T-cell responses.

Although early clinical studies demonstrated measurable immunogenicity, objective clinical responses were generally modest. One major limitation involved antigen heterogeneity, as variable antigen expression among tumor clones facilitated immune escape through the selective outgrowth of antigen-negative populations.

In addition, many shared tumor-associated antigens represent self-antigens and are therefore subject to central immune tolerance mechanisms that limit vaccine-induced immune responsiveness. These findings reinforce the limitations of isolated single-antigen vaccine approaches and support the development of broader multi-antigen and personalized neoantigen-based strategies.

### 6.4. Post-Transplant and Minimal Residual Disease-Directed Vaccination Strategies

Among the clinical settings evaluated in multiple myeloma, the post-autologous stem cell transplantation (ASCT) and minimal residual disease (MRD) settings have emerged as some of the most favorable environments for therapeutic vaccination. High-dose chemotherapy substantially reduces tumor burden, while subsequent immune reconstitution creates a biologic milieu characterized by increased homeostatic cytokine signaling and enhanced lymphocyte expansion. These conditions may facilitate more effective vaccine-induced T-cell priming, expansion, and persistence compared with heavily pretreated or progressive disease states [45,47,51].

The degree of tumor burden appears to profoundly influence vaccine efficacy. High tumor burden promotes establishment of an immunosuppressive tumor microenvironment characterized by inhibitory cytokine signaling, impaired dendritic cell function, regulatory T-cell expansion, and progressive T-cell exhaustion. In contrast, MRD settings provide more favorable biologic conditions for vaccine-induced immune activation and maintenance of antitumor immune surveillance.

Several studies demonstrated that vaccination following ASCT could induce durable tumor-specific immune responses together with prolonged disease stabilization [45,47]. Increasingly, MRD-directed vaccination strategies are being explored as consolidation or maintenance approaches aimed at delaying relapse through sustained immune-mediated control of residual malignant clones.

Biavati and colleagues evaluated a granulocyte–macrophage colony-stimulating factor (GM-CSF)-secreting allogeneic myeloma vaccine (GVAX) combined with lenalidomide in patients with minimal residual disease and observed deep immune responses together with prolonged progression-free survival in selected patients [48]. These findings suggest potential synergistic effects between therapeutic vaccination and immunomodulatory therapy in low-disease-burden settings.

In contrast, Qazilbash and colleagues evaluated idiotype vaccination combined with adoptive autologous T-cell transfer and demonstrated strong immune activation without significant improvement in progression-free survival [49]. These observations highlight both the promise and limitations of therapeutic cancer vaccination in multiple myeloma while reinforcing the importance of multi-antigen targeting, immune modulation, and rational combination immunotherapy strategies.

Collectively, these findings contributed to a broader conceptual shift in therapeutic vaccine development. Rather than functioning primarily as direct cytotoxic therapies against bulky active disease, therapeutic vaccines may ultimately prove most effective as consolidation or maintenance strategies designed to sustain long-term immune surveillance during periods of minimal residual disease.

### 6.5. Combination Immunotherapy Approaches

Increasing evidence suggests that vaccine monotherapy is insufficient to overcome the complex immunosuppressive environment characteristic of multiple myeloma. Consequently, substantial interest has focused on rational combination strategies designed to augment vaccine efficacy through the simultaneous enhancement of immune activation and reversal of tumor-mediated immune suppression.

Immunomodulatory drugs such as lenalidomide and pomalidomide enhance T-cell and natural killer cell activity, reduce regulatory T-cell populations, and improve dendritic cell function, thereby providing a strong biologic rationale for combination with therapeutic vaccination [48]. Early clinical studies combining vaccines with lenalidomide demonstrated enhanced antigen-specific immune responses and improved T-cell activation, particularly in low-disease-burden settings.

Checkpoint inhibition represents another important strategy because vaccine-induced T-cell activation frequently becomes limited by PD-1-mediated exhaustion and other inhibitory signaling pathways [16,17,18,19]. Although checkpoint inhibitors demonstrated limited efficacy as monotherapy in multiple myeloma, their integration with therapeutic vaccines may enhance persistence and functionality of vaccine-induced T-cell responses, particularly when administered during periods of minimal residual disease.

Additional investigational approaches include combining vaccines with radiation therapy, epigenetic modulators, monoclonal antibodies, bispecific antibodies, and CAR T-cell therapy. These strategies aim to simultaneously improve antigen presentation, reduce immune suppression, broaden immune targeting, and enhance long-term immune surveillance.

Emerging concepts additionally include post-CAR T-cell vaccination approaches intended to sustain immune control following deep responses achieved through cellular therapy. Vaccination in this setting may potentially broaden antigenic targeting beyond the primary CAR target and reduce relapse risk associated with antigen escape and clonal evolution.

### 6.6. Neoantigen and mRNA Vaccine Approaches

Recent advances in next-generation sequencing, computational neoantigen prediction, and mRNA vaccine technology have substantially renewed interest in personalized vaccine strategies for multiple myeloma [20,21,22,23,24,25,26].

Ongoing somatic evolution and clonal heterogeneity generate potentially immunogenic neoantigens that may be targeted through individualized vaccine approaches. Unlike shared tumor-associated antigens, neoantigens are absent from normal tissues and therefore may induce stronger and more tumor-specific immune responses.

mRNA-based vaccine platforms are particularly attractive because they permit the rapid incorporation of multiple neoantigen targets while supporting scalable manufacturing and adaptive vaccine modification. These technologies additionally enable the dynamic adjustment of vaccine composition in response to tumor evolution and the emergence of resistant subclones.

Recent clinical studies involving personalized mRNA neoantigen vaccines demonstrated durable T-cell responses and encouraging clinical activity, particularly when combined with checkpoint inhibition [31,41,44,52]. Although clinical experience in multiple myeloma remains limited, these findings have significantly accelerated interest in applying mRNA vaccine technology within hematologic malignancies.

Importantly, these advances support a broader transition from static single-antigen vaccine models toward adaptive and precision-based immunotherapeutic strategies capable of dynamically responding to tumor evolution and immune escape mechanisms.

### 6.7. Translational Challenges and Future Directions

Despite encouraging advances, several important challenges continue to limit the widespread clinical implementation of therapeutic cancer vaccines in multiple myeloma. Persistent immune suppression within the bone marrow microenvironment, tumor heterogeneity, antigenic drift, and progressive T-cell exhaustion remain major barriers to durable immune-mediated disease control [16,17,18,19].

In addition, personalized vaccine approaches require efficient neoantigen identification, the accurate prediction of immunogenic epitopes, scalable manufacturing, and rapid production timelines. Current computational algorithms remain imperfect, and not all predicted neoantigens generate clinically meaningful immune responses.

Future progress will likely depend upon improved biomarker-driven patient selection, advanced immune monitoring, longitudinal genomic profiling, and rational integration with complementary immunotherapies. Minimal residual disease assessment, T-cell repertoire analysis, and dynamic monitoring of tumor evolution may facilitate increasingly adaptive and individualized treatment strategies.

Collectively, these developments support a major conceptual transition in multiple myeloma immunotherapy—from relatively static single-antigen vaccine models toward integrated precision immuno-oncology approaches designed to establish durable immune surveillance and long-term disease control [28,29,33,41,42,43,44,51,52].

## 7. Combination Immunotherapy

One of the most important lessons derived from early therapeutic vaccine studies is that vaccine monotherapy is frequently insufficient to overcome the complex immunosuppressive environment present in established malignancies. Although many vaccine platforms successfully generated measurable antigen-specific immune responses, durable clinical benefit remained inconsistent because activated T cells often became functionally suppressed within the tumor microenvironment. Consequently, modern vaccine development increasingly emphasizes rational combination strategies designed to simultaneously enhance immune activation and reverse tumor-mediated immunosuppression.

Combination immunotherapy seeks to optimize multiple steps of the cancer–immunity cycle simultaneously, including antigen release, dendritic cell activation, T-cell priming, the trafficking of effector cells into tumor sites, and maintenance of durable immune memory. These integrated approaches increasingly represent the future direction of therapeutic cancer vaccination.

### 7.1. Checkpoint Inhibition

Immune checkpoint pathways, including PD-1/PD-L1 and CTLA-4, play critical roles in regulating T-cell activation and maintaining immune homeostasis [16,17,18,19]. Tumors frequently exploit these inhibitory pathways to induce T-cell exhaustion and suppress antitumor immune responses. Checkpoint inhibitors restore T-cell activity by blocking these suppressive signals, thereby enhancing proliferation, cytokine production, and cytotoxic function.

The combination of therapeutic vaccination with checkpoint blockade provides a strong mechanistic rationale. Vaccines expand tumor-specific T-cell populations, while checkpoint inhibitors preserve and augment their functional activity within the tumor microenvironment. Clinical studies in solid tumors have demonstrated improved T-cell expansion, enhanced intratumoral immune infiltration, and superior clinical outcomes when neoantigen or mRNA-based vaccines are combined with checkpoint inhibition [41,42,43,44]. Early studies in hematologic malignancies, particularly in post-transplant or MRD settings, similarly suggest that checkpoint blockade may deepen responses and improve the persistence of vaccine-induced immunity [47,48,52].

### 7.2. Immunomodulatory Agents

Immunomodulatory drugs (IMiDs), particularly lenalidomide, exert broad stimulatory effects on both innate and adaptive immunity. These agents enhance T-cell and natural killer (NK) cell function, improve immune synapse formation, increase cytokine production, and reduce suppressive regulatory T-cell populations [48,62].

Within the context of therapeutic vaccination, IMiDs may augment immune priming while simultaneously reducing immunosuppressive signaling pathways. Clinical studies in multiple myeloma have demonstrated that combining vaccines with lenalidomide can enhance immune responses and may improve progression-free survival in selected patients [48]. These findings support the incorporation of immunomodulatory agents into combination vaccine strategies, particularly in maintenance or MRD settings.

### 7.3. Chemotherapy and Radiation Therapy

Conventional cancer therapies may also exert important immunomodulatory effects capable of enhancing vaccine efficacy. Radiation therapy promotes immunogenic tumor-cell death, increases tumor antigen release, enhances dendritic cell activation, and may increase MHC expression on malignant cells. These effects can augment antigen presentation and facilitate epitope spreading, thereby amplifying vaccine-induced immune responses.

Similarly, selected chemotherapeutic agents may reduce immunosuppressive cell populations including regulatory T cells and myeloid-derived suppressor cells while simultaneously decreasing tumor burden. Reduced disease burden may create a more favorable environment for effective immune priming and T-cell expansion.

These observations have led to increasing interest in integrating therapeutic vaccines into multimodality treatment strategies rather than administering them as isolated interventions. In particular, post-cytoreductive minimal residual disease settings appear especially favorable because the lower tumor burden may permit more effective immune-mediated disease control.

### 7.4. Epigenetic Modulation

Epigenetic therapies including hypomethylating agents and histone deacetylase inhibitors may enhance tumor immunogenicity through upregulation of tumor-associated antigens, MHC molecules, and co-stimulatory signaling pathways.

In hematologic malignancies, epigenetic modulation may increase the expression of cancer-testis antigens such as NY-ESO-1 and MAGE family proteins, thereby improving recognition by vaccine-induced T cells. These findings provide a mechanistic rationale for combining therapeutic vaccination with epigenetic therapies to overcome immune escape associated with reduced antigen expression.

Although clinical experience remains limited, this strategy represents a promising avenue for future translational investigation.

### 7.5. Cellular Therapies and Post-CAR T-Cell Vaccination

The rapid emergence of CAR T-cell therapy and bispecific antibodies has transformed treatment paradigms in B-cell malignancies and multiple myeloma. These therapies can induce deep remissions and minimal residual disease-negative states even in heavily pretreated patients.

Importantly, vaccine strategies may complement cellular therapies by sustaining long-term immune surveillance following initial cytoreduction. CAR T-cell therapy frequently produces profound reductions in tumor burden but may not fully prevent subsequent relapse because of antigen escape, limited persistence, or progressive immune dysfunction.

Vaccination following CAR T-cell therapy therefore represents an emerging concept aimed at maintaining durable immune control against residual tumor clones. In this setting, vaccines may broaden antigenic targeting beyond the primary CAR target and reduce the likelihood of relapse through antigen escape mechanisms.

Similarly, bispecific antibodies capable of redirecting endogenous T cells toward malignant cells may synergize with vaccines by enhancing T-cell activation and tumor-cell killing. These approaches remain investigational but represent important future directions in integrated immunotherapy.

### 7.6. MRD as an Immunologic Window

Accumulating evidence suggests that minimal residual disease (MRD) states provide one of the most favorable biologic settings for therapeutic vaccination. Reduced tumor burden decreases overall immunosuppressive signaling, while recovering immune populations following therapy may facilitate more effective antigen presentation and T-cell expansion.

This concept is particularly relevant following autologous stem cell transplantation, CAR T-cell therapy, and other highly cytoreductive treatments. Rather than functioning primarily as direct cytotoxic therapies against bulky disease, vaccines may be more effective as maintenance or consolidation strategies designed to sustain long-term immune surveillance and prevent relapse.

Accordingly, future clinical trials will likely increasingly incorporate MRD-guided treatment strategies together with advanced immune monitoring to identify optimal timing for therapeutic vaccination.

### 7.7. Synthesis and Future Perspectives

Collectively, current evidence supports a major conceptual shift in therapeutic cancer vaccination. Earlier vaccine strategies frequently focused on the isolated induction of antigen-specific immunity; however, modern approaches increasingly recognize that successful antitumor immunity requires coordinated modulation of multiple immune pathways simultaneously.

Consequently, therapeutic vaccines are unlikely to achieve maximal efficacy as standalone interventions. Instead, their future role will likely involve integration into broader immunotherapeutic frameworks combining vaccination with checkpoint inhibition, immunomodulatory agents, epigenetic therapies, radiation therapy, chemotherapy, and cellular therapies [16,17,18,19,38,39,40,41,42,43,44,47,48,51,52].

This integrated strategy reflects an evolving understanding that durable tumor control depends not solely on immune activation but also on maintenance of functional immune persistence, prevention of immune escape, and continuous adaptation to tumor evolution.

## 8. Challenges and Future Directions

Despite substantial advances in cancer immunotherapy, therapeutic cancer vaccines have historically produced inconsistent clinical outcomes in B-cell malignancies and multiple myeloma. Early studies clearly demonstrated the ability to induce tumor-specific immune responses; however, durable and reproducible clinical benefit remained limited in many settings. These observations highlight the biologic complexity of antitumor immunity and underscore the need for more sophisticated and integrated vaccine strategies.

Multiple barriers contribute to these limitations, including tumor heterogeneity, immune escape, impaired antigen presentation, T-cell exhaustion, and logistical challenges associated with personalized vaccine production. Understanding these challenges is essential for the development of next-generation therapeutic vaccination approaches.

### 8.1. Tumor Heterogeneity and Immune Escape

Tumor heterogeneity remains one of the most significant barriers to effective vaccine therapy. Malignant cell populations continually evolve under selective immune and therapeutic pressure, resulting in dynamic changes in antigen expression and clonal composition [5,6].

Earlier vaccine approaches frequently targeted single antigens such as idiotype proteins or individual tumor-associated antigens. Although these strategies successfully induced antigen-specific immune responses, tumors often escaped immune control through antigen loss, downregulation of antigen presentation machinery, or expansion of antigen-negative subclones [16,17,18,19].

This phenomenon is particularly relevant in multiple myeloma and aggressive lymphomas, where ongoing genomic instability and clonal evolution generate substantial intratumoral heterogeneity. These observations strongly support the development of multi-antigen and personalized neoantigen-based vaccine strategies capable of targeting broader repertoires of tumor-specific epitopes [20,21,22].

Epitope spreading may partially compensate for antigenic heterogeneity by allowing immune responses to expand beyond initially targeted antigens. However, this process is often insufficient to fully prevent immune escape in advanced disease.

### 8.2. Immunosuppressive Tumor Microenvironment

The tumor microenvironment exerts profound inhibitory effects on antitumor immunity and represents a major obstacle to successful therapeutic vaccination. Malignant cells and associated stromal populations secrete immunosuppressive cytokines including transforming growth factor-β, interleukin-10, and vascular endothelial growth factor, all of which impair dendritic cell maturation and suppress T-cell activation [16,17,18,19].

In addition, tumors recruit regulatory T cells, tumor-associated macrophages, and myeloid-derived suppressor cells that further inhibit immune responses through multiple complementary pathways [37]. Persistent antigen exposure additionally promotes T-cell exhaustion characterized by impaired proliferation, reduced cytokine production, and diminished cytotoxic activity.

These mechanisms collectively explain why vaccine-induced immune responses frequently fail to translate into durable clinical benefit despite measurable immunogenicity. Consequently, modern therapeutic strategies increasingly focus on combining vaccination with checkpoint inhibition, immunomodulatory agents, or other approaches capable of reversing immune suppression within the tumor microenvironment.

### 8.3. Limitations of Early Idiotype Vaccine Strategies

Idiotype vaccines represented a major conceptual milestone in personalized cancer immunotherapy and provided important proof-of-principle evidence that tumor-specific immune responses could be induced in hematologic malignancies [10,24,25]. However, several limitations prevented their widespread clinical implementation.

First, idiotype antigens often demonstrated relatively weak intrinsic immunogenicity, necessitating the use of carrier proteins, adjuvants, or dendritic cell platforms to generate effective immune responses. Second, individualized manufacturing was labor-intensive, expensive, and associated with prolonged production timelines that frequently delayed treatment initiation.

The Phase III follicular lymphoma trial conducted by Schuster and colleagues highlighted these logistical challenges, as many enrolled patients were unable to receive vaccination because of manufacturing delays or disease progression before vaccine availability [54].

Importantly, these limitations reflected practical and translational barriers rather than absence of biologic activity. Indeed, vaccinated patients frequently demonstrated improved disease-free survival and durable immune responses, thereby supporting the underlying therapeutic rationale.

These experiences have substantially informed the development of modern vaccine technologies emphasizing scalable manufacturing, rapid production, and multi-antigen targeting.

### 8.4. Challenges in Neoantigen Identification

Personalized neoantigen vaccines represent one of the most promising advances in therapeutic cancer vaccination; however, several important technical challenges remain [20,21,22].

Neoantigen discovery requires high-quality tumor sequencing, accurate mutation calling, prediction of MHC-binding affinity, and identification of immunogenic epitopes capable of generating meaningful T-cell responses. Current computational algorithms remain imperfect, and predicted neoantigens do not always translate into effective immune targets in vivo.

In addition, tumor evolution may alter neoantigen expression over time, thereby reducing vaccine relevance if substantial delays occur between sequencing and treatment. These challenges underscore the need for adaptive vaccine platforms capable of rapidly incorporating newly emerging tumor mutations.

Advances in machine learning, computational immunology, and sequencing technologies are progressively improving neoantigen prediction accuracy and reducing production timelines, thereby increasing clinical feasibility.

### 8.5. Biomarker Development and Immune Monitoring

A major limitation of earlier vaccine studies involved inadequate biomarkers capable of predicting response or guiding patient selection. Traditional clinical endpoints such as tumor shrinkage may incompletely capture the delayed kinetics and prolonged immune-mediated effects characteristic of vaccine therapy.

Future progress will likely depend on the integration of advanced immune monitoring techniques including T-cell receptor sequencing, cytokine profiling, immune repertoire analysis, minimal residual disease assessment, and longitudinal genomic monitoring of tumor evolution.

These technologies may facilitate the identification of patients most likely to benefit from therapeutic vaccination while additionally permitting the adaptive modification of treatment strategies based on evolving immune and tumor dynamics.

### 8.6. mRNA Technology and Next-Generation Vaccine Platforms

The rapid success of mRNA vaccine technology has substantially accelerated interest in therapeutic cancer vaccination [24,25,26]. mRNA platforms offer several important advantages including rapid manufacturing, scalability, flexibility, and the ability to encode multiple antigens simultaneously.

Importantly, mRNA vaccines support increasingly personalized and adaptive therapeutic strategies because vaccine composition can be rapidly modified in response to evolving tumor genomics. These platforms additionally avoid risks associated with genomic integration and possess intrinsic immunostimulatory properties that enhance dendritic cell activation.

Recent clinical studies involving personalized neoantigen mRNA vaccines in melanoma demonstrated robust and durable T-cell responses together with encouraging clinical efficacy, particularly when combined with checkpoint inhibition [36,41,44,52]. Although experience in hematologic malignancies remains limited, these advances represent one of the most promising future directions in the field.

### 8.7. Future Conceptual Framework

An important conceptual shift emerging from recent advances is the transition away from static single-antigen vaccine models toward dynamic and integrated immunotherapeutic strategies. Successful therapeutic vaccination likely depends on the coordinated optimization of multiple interconnected variables, including antigen selection, scalable and adaptive delivery platforms, biologically favorable treatment timing, reversal of tumor-mediated immune suppression, integration with complementary immunotherapies, and longitudinal immune monitoring.

In this evolving framework, therapeutic vaccines may function less as isolated cytotoxic therapies and more as modulators of long-term immune surveillance capable of maintaining durable control over residual disease.

Minimal residual disease settings following cytoreductive therapy, autologous stem cell transplantation, CAR T-cell therapy, or bispecific antibody treatment may provide particularly favorable opportunities for vaccine integration because of reduced tumor burden and partial immune reconstitution.

The field now stands at a point where precision oncology and immunotherapy are increasingly able to integrate genomic information, immune profiling, and sophisticated vaccine methodologies to enable highly individualized therapeutic approaches [34,35,36,37,38,39,40,41,42,43,44,51,52]. While obstacles including tumor heterogeneity, immune evasion, and practical manufacturing scalability persist, progress to date provides a strong foundation for advancing therapeutic cancer vaccines toward precision immuno-oncology [5,6,16,17,18,19,35,54]. These approaches aim beyond temporary immune activation to establish durable immune surveillance capable of enabling long-term disease control [7,36,41].

## 9. Clinical Perspective

Therapeutic cancer vaccination in B-cell malignancies and multiple myeloma has evolved from early empirical idiotype strategies toward increasingly precise, mechanistically informed immunotherapeutic approaches. Early studies in follicular lymphoma demonstrated that tumor-specific immune responses can be generated in patients with established malignancy and that vaccine-induced T-cell responses may correlate with clinical outcomes [10,25,53,55,56,57,59]. These investigations established important proof-of-principle observations that continue to guide modern vaccine development. Accordingly, the present review emphasizes evolving translational frameworks and emerging immunotherapeutic strategies in addition to currently available clinical evidence.

A central insight emerging from these studies is that vaccine efficacy is highly context dependent. High tumor burden promotes profound immunosuppression through expansion of regulatory immune cell populations, inhibitory cytokine signaling, and activation of immune checkpoint pathways. In contrast, minimal residual disease (MRD) settings provide more favorable immunologic environments characterized by lower tumor burden and partial immune reconstitution, thereby enhancing dendritic cell function, T-cell priming, and immune expansion. Clinical studies in both follicular lymphoma and multiple myeloma suggest that therapeutic vaccination may achieve the greatest efficacy in MRD settings, supporting its development as a consolidation or maintenance strategy rather than as a treatment for overt bulky disease [25,48,51,54].

T-cell-mediated immunity appears central to effective therapeutic vaccination. Cellular immune responses, particularly those involving cytotoxic CD8^+^ T lymphocytes, correlate more consistently with clinical outcomes than humoral immune responses alone. The generation of durable memory T-cell populations capable of sustained tumor surveillance therefore represents a major objective of modern vaccine design and influences both antigen selection and platform development.

Antigen selection strategies have evolved substantially over time—from idiotype antigens, which possess high tumor specificity but substantial manufacturing limitations, to shared tumor-associated antigens with broader applicability but increased susceptibility to immune tolerance and antigenic heterogeneity, and more recently toward personalized neoantigen approaches characterized by improved tumor specificity and immunogenicity [15,34,35,36].

Recent clinical experience suggests that neoantigen-based vaccine strategies, particularly when delivered through mRNA-based platforms and combined with checkpoint inhibition, represent one of the most promising directions in therapeutic cancer vaccination. However, much of the currently available clinical evidence derives from solid tumors, and experience in hematologic malignancies remains comparatively limited.

mRNA-based vaccine platforms address several important limitations associated with earlier technologies by enabling rapid manufacturing, simultaneous multi-antigen encoding, scalable production, and efficient intracellular antigen delivery [38,39,40]. These features support increasingly adaptive and personalized immunotherapeutic approaches capable of responding dynamically to tumor evolution and immune escape mechanisms.

Importantly, accumulating evidence indicates that vaccine monotherapy is unlikely to achieve meaningful clinical benefit in most settings. Rational combination strategies incorporating checkpoint inhibitors, immunomodulatory agents, radiation therapy, epigenetic therapies, and cellular immunotherapies will likely be required to overcome tumor-mediated immune suppression and sustain durable antitumor immunity.

The development of therapeutic cancer vaccines additionally highlights the need for refined clinical endpoints and biomarker-driven treatment strategies. Conventional endpoints such as progression-free survival may incompletely capture the delayed yet durable effects of immunotherapy. Alternative approaches incorporating minimal residual disease assessment, immune correlates, longitudinal immune profiling, and overall survival may provide more comprehensive measures of therapeutic efficacy.

Taken together, these observations support an ongoing conceptual transition toward precision immuno-oncology, in which vaccine strategies are tailored according to disease biology, immune context, treatment timing, and dynamic tumor evolution. The integration of genomic profiling, immune monitoring, and advanced vaccine platforms increasingly enables personalized and adaptive therapeutic approaches.

Although significant challenges remain—including tumor heterogeneity, immune evasion, and manufacturing complexity—progress achieved to date provides a strong foundation for the continued advancement of therapeutic cancer vaccines. The emerging paradigm increasingly emphasizes integrated immunotherapeutic strategies combining vaccination with complementary treatment modalities to establish durable immune surveillance and long-term disease control.

## 10. Conclusions

Therapeutic cancer vaccines represent an important and evolving component of modern immuno-oncology. Early studies in B-cell malignancies and multiple myeloma established proof-of-principle that tumor-specific immune responses can be generated through vaccination strategies targeting idiotype proteins, shared tumor-associated antigens, and dendritic cell-based platforms [10,24,25,45,46]. Although many early approaches demonstrated measurable immunogenicity, durable clinical benefit remained inconsistent because of tumor heterogeneity, immune escape mechanisms, weak antigen immunogenicity, impaired antigen presentation, and highly suppressive tumor microenvironments [16,17,18,19].

Despite these challenges, lessons learned from earlier vaccine studies have substantially informed the development of next-generation immunotherapeutic strategies. Advances in next-generation sequencing, computational neoantigen prediction, immune profiling, and scalable vaccine manufacturing technologies have transformed the field from relatively static single-antigen approaches toward increasingly adaptive and personalized therapeutic platforms [34,35,36,37,38,39,40].

In particular, neoantigen vaccines and mRNA-based technologies have renewed enthusiasm for therapeutic cancer vaccination by enabling the rapid and individualized targeting of tumor-specific mutations while simultaneously supporting scalable production and dynamic adaptation to tumor evolution [38,39,40,41,42,43,44,51,52]. These approaches may substantially reduce immune escape associated with antigen loss and clonal heterogeneity while improving the breadth and durability of vaccine-induced immune responses.

Contemporary evidence increasingly suggests that therapeutic vaccines are unlikely to achieve maximal efficacy as isolated interventions and will likely require integration with complementary immunotherapeutic strategies including checkpoint inhibition, immunomodulatory agents, and cellular therapies [16,17,18,19,47,48].

Future progress will depend upon improved neoantigen identification algorithms, advanced immune monitoring, biomarker-driven patient selection, scalable manufacturing platforms, and rational integration with complementary immunotherapies. Longitudinal genomic and immune profiling may further enable adaptive vaccine modification in response to evolving tumor biology and immune dynamics.

While current clinical evidence in B-cell malignancies remains relatively early-stage compared with solid-tumor vaccine development, ongoing advances in precision immunotherapy and adaptive vaccine technologies continue to expand the translational potential of therapeutic cancer vaccination. Although substantial challenges including tumor heterogeneity, immune evasion, and manufacturing complexity remain, the progress achieved to date provides a strong foundation for advancing therapeutic cancer vaccines toward precision immuno-oncology [5,6,16,17,18,19,35,54]. These approaches increasingly aim beyond transient immune activation to establish durable immune surveillance and long-term disease control [7,36,41].

## Figures and Tables

**Table 1:** Major Tumor Antigens and Vaccine Targets in B-Cell Malignancies and Multiple Myeloma.

Antigen Category	Representative Antigens	Advantages	Limitations	Representative Malignancies
Idiotype antigens	Patient-specific immunoglobulin variable regions	Highly tumor-specific; individualized targeting	Weak intrinsic immunogenicity; complex manufacturing	Follicular lymphoma, multiple myeloma
Lineage-associated antigens	CD19, CD20, CD22, CD38, BCMA	Broad expression within malignant lineage; established therapeutic relevance	Off-tumor effects on normal B cells/plasma cells	B-cell lymphomas, multiple myeloma
Cancer-testis antigens	NY-ESO-1, MAGE-A3, PRAME	High immunogenicity; restricted normal tissue expression	Variable expression; immune escape	Multiple myeloma, lymphoma
Differentiation antigens	WT1, survivin	Shared tumor-associated targets; broad applicability	Central immune tolerance; lower specificity	Leukemia, lymphoma, multiple myeloma
Neoantigens	Mutation-derived patient-specific epitopes	Highly tumor-specific; reduced immune tolerance	Requires genomic sequencing and computational prediction	Multiple hematologic malignancies
Whole tumor antigens	Tumor lysates/apoptotic tumor cells	Broad antigen repertoire; reduced antigen escape	Variable antigen composition; less standardized	Multiple hematologic malignancies

**Table 2:** Therapeutic Vaccine Platforms and Translational Characteristics in B-Cell Malignancies and Multiple Myeloma.

Vaccine Platform	Mechanism of Immune Activation	Advantages	Limitations	Representative Applications
Idiotype protein vaccines	Patient-specific idiotype proteins administered with carrier proteins/adjuvants stimulate antigen-specific T-cell and humoral responses	Highly individualized and tumor-specific	Complex manufacturing; prolonged production timelines; variable immunogenicity	Follicular lymphoma, multiple myeloma
Dendritic cell vaccines	Ex vivo antigen-loaded dendritic cells activate CD4^+^ and CD8^+^ T-cell responses	Potent antigen presentation and cellular immune activation	Labor-intensive preparation; variable standardization	Multiple myeloma, lymphoma
Peptide-based vaccines	Synthetic tumor-associated antigen peptides presented through MHC pathways	Relatively simple manufacturing; defined antigen specificity	HLA restriction; weaker immunogenicity	NY-ESO-1, WT1, survivin-targeted vaccines
Whole tumor-cell vaccines	Irradiated autologous or allogeneic tumor cells provide broad antigen exposure	Broad antigen repertoire with reduced single-antigen escape	Limited standardization; inconsistent immune responses	GVAX approaches
Viral vector vaccines	Viral-mediated intracellular delivery of tumor antigens enhances immune stimulation	Strong innate and adaptive immune activation	Pre-existing antiviral immunity; vector-related toxicity concerns	Investigational hematologic vaccine platforms
DNA vaccines	Plasmid DNA encoding tumor antigens induces endogenous antigen expression	Stable and scalable manufacturing	Relatively limited immunogenicity in humans	Experimental studies
mRNA vaccines	Lipid nanoparticle-delivered mRNA encoding tumor antigens promotes intracellular antigen expression	Rapid manufacturing; multi-antigen targeting; scalable and adaptable platforms	Cold-chain and delivery-related logistical challenges	Personalized neoantigen vaccines
Neoantigen vaccines	Patient-specific mutation-derived epitopes induce individualized T-cell responses	Highly tumor-specific with reduced immune tolerance	Requires genomic sequencing and bioinformatic prediction	Precision immuno-oncology strategies

**Table 3:** Selected Clinical Trials of Therapeutic Cancer Vaccines in B-Cell Malignancies and Multiple Myeloma, with selected solid tumor studies providing proof-of-concept for neoantigen vaccine approaches.

Study/Year	Disease	Vaccine Type	Target Antigen(s)	Setting	Combination	Key Outcomes
Kwak et al., 1992 [10]	NHL	Idiotype protein (KLH-conjugated)	Idiotype	MRD	None	Immune responses; proof-of-concept
Hsu et al., 1996 [46]	NHL	DC-based	Idiotype	MRD	None	Immune activation with clinical responses
Bendandi et al., 1999 [25]	FL	Idiotype + GM-CSF	Idiotype	MRD	GM-CSF	Molecular remissions in subset
Timmerman et al., 2002 [53]	FL	DC-based	Idiotype	Active disease	None	Tumor regression; booster effect
Schuster et al., 2011 [54]	FL	Patient-specific vaccine	Idiotype	CR1	None	Improved DFS in treated patients
Rosenblatt et al., 2011 [45]	MM	DC–tumor fusion	Multiple antigens	Relapsed	None	Immune responses; stabilization
Rosenblatt et al., 2013 [47]	MM	DC–tumor fusion	Multiple antigens	Post-ASCT	Anti–PD-1	Enhanced responses
Biavati et al., 2021 [48]	MM	GVAX	Whole tumor	MRD	Lenalidomide	Prolonged PFS
Freeman et al., 2023 [52]	MM	DC vaccine	Survivin	Post-induction	None	Durable responses
Ott et al., 2020 [41]	Solid tumors	Neoantigen peptide	Personalized neoAgs	Adjuvant	None	Robust T-cell responses
Sahin et al., 2017 [36]	Solid tumors	mRNA neoantigen	Personalized neoAgs	Adjuvant	None	Durable immunity
Keskin et al., 2019 [42]	GBM	Neoantigen peptide	Personalized neoAgs	Adjuvant	None	Intratumoral T-cell infiltration
Hilf et al., 2019 [43]	GBM	Multi-peptide	Tumor + neoAgs	Adjuvant	None	Immune responses
mRNA-4157 + Pembrolizumab, 2024 [44]	Melanoma	mRNA neoantigen	Personalized neoAgs	Adjuvant	PD-1 inhibitor	Improved RFS

## Data Availability

No new data were generated or analyzed in this study. Data sharing is not applicable to this article.
